# Supplementary material for: Hot spot prediction in protein-protein interactions by an ensemble system
Source: BMC Syst Biol. 2018 Dec 31;12(Suppl 9):132. doi: 10.1186/s12918-018-0665-8 (PMC6311905; doi:10.1186/s12918-018-0665-8)
Supplement: Supplementary file 2 — All relevant MATLAB code. A simple Matlab implement of our predictor. (ZIP 19,742 kb) [file 12918_2018_665_MOESM2_ESM.zip › Additional file 2/Read ASA/ASA website/SKEMPI/2VLJD.htm]

 NetSurfP - Protein Surface Accessibility and Secondary Structure Predictions

  

|  |  |  |
| --- | --- | --- |
|  |  | NetSurfP - Protein Surface Accessibility and Secondary Structure Predictions  Technical University of Denmark |

  


---

```
# For publication of results, please cite:
# A generic method for assignment of reliability scores applied to solvent accessibility predictions.
# Bent Petersen, Thomas Nordahl Petersen, Pernille Andersen, Morten Nielsen and Claus Lundegaard
# BMC Structural Biology 2009, 9:51 doi:10.1186/1472-6807-9-51
#
# Column 1: Class assignment - B for buried or E for Exposed - Threshold: 25% exposure, but not based on RSA
# Column 2: Amino acid
# Column 3: Sequence name
# Column 4: Amino acid number
# Column 5: Relative Surface Accessibility - RSA
# Column 6: Absolute Surface Accessibility
# Column 7: Z-fit score for RSA prediction
# Column 8: Probability for Alpha-Helix
# Column 9: Probability for Beta-strand
# Column 10: Probability for Coil
E Q  2VLJD                  1    0.564 100.766  -0.322   0.003   0.003   0.994
E L  2VLJD                  2    0.604 110.519   1.260   0.004   0.514   0.481
B L  2VLJD                  3    0.158  28.930  -0.387   0.004   0.616   0.381
E E  2VLJD                  4    0.520  90.861   0.162   0.004   0.616   0.381
B Q  2VLJD                  5    0.196  34.988  -0.754   0.005   0.336   0.660
E S  2VLJD                  6    0.416  48.779   0.217   0.004   0.085   0.910
E P  2VLJD                  7    0.509  72.156  -0.823   0.018   0.019   0.964
E Q  2VLJD                  8    0.581 103.731  -1.103   0.018   0.088   0.893
E F  2VLJD                  9    0.382  76.708  -1.049   0.021   0.451   0.528
B L  2VLJD                 10    0.234  42.919   0.056   0.021   0.756   0.223
E S  2VLJD                 11    0.450  52.763  -0.396   0.018   0.846   0.136
B I  2VLJD                 12    0.255  47.193   0.387   0.023   0.655   0.322
E Q  2VLJD                 13    0.443  79.048   0.690   0.004   0.420   0.576
E E  2VLJD                 14    0.547  95.491   0.598   0.004   0.085   0.910
E G  2VLJD                 15    0.662  52.107  -0.952   0.005   0.015   0.979
E E  2VLJD                 16    0.489  85.341   0.549   0.004   0.138   0.858
E N  2VLJD                 17    0.582  85.190   1.250   0.003   0.718   0.279
B L  2VLJD                 18    0.086  15.728   0.612   0.001   0.959   0.040
E T  2VLJD                 19    0.416  57.741   1.192   0.001   0.959   0.040
B V  2VLJD                 20    0.057   8.807  -0.009   0.001   0.959   0.040
E Y  2VLJD                 21    0.409  87.339   0.797   0.001   0.959   0.040
B C  2VLJD                 22    0.038   5.293  -0.606   0.001   0.900   0.099
E N  2VLJD                 23    0.419  61.342   0.960   0.001   0.900   0.099
E S  2VLJD                 24    0.327  38.324  -1.354   0.002   0.816   0.182
E S  2VLJD                 25    0.377  44.243  -1.330   0.004   0.514   0.481
E S  2VLJD                 26    0.496  58.096  -1.931   0.004   0.085   0.910
E V  2VLJD                 27    0.456  70.133  -2.262   0.018   0.047   0.935
E F  2VLJD                 28    0.304  61.093  -0.575   0.018   0.088   0.893
B S  2VLJD                 29    0.283  33.132  -0.869   0.020   0.205   0.775
B S  2VLJD                 30    0.139  16.314   0.321   0.022   0.552   0.426
B L  2VLJD                 31    0.028   5.090   0.470   0.021   0.756   0.223
B Q  2VLJD                 32    0.093  16.592   0.345   0.072   0.855   0.073
B W  2VLJD                 33    0.032   7.600   0.231   0.072   0.855   0.073
B Y  2VLJD                 34    0.043   9.104   0.662   0.072   0.855   0.073
B R  2VLJD                 35    0.159  36.297   0.738   0.085   0.775   0.140
B Q  2VLJD                 36    0.095  16.896   0.709   0.079   0.592   0.329
E E  2VLJD                 37    0.367  64.115   1.690   0.018   0.088   0.893
E P  2VLJD                 38    0.389  55.256   0.207   0.005   0.015   0.979
E G  2VLJD                 39    0.833  65.589  -1.824   0.003   0.003   0.994
E E  2VLJD                 40    0.432  75.401   0.326   0.018   0.019   0.964
E G  2VLJD                 41    0.409  32.220   0.142   0.018   0.047   0.935
B P  2VLJD                 42    0.084  11.920   0.733   0.021   0.279   0.699
B V  2VLJD                 43    0.246  37.872   0.551   0.018   0.846   0.136
B L  2VLJD                 44    0.107  19.628   0.558   0.011   0.918   0.071
B L  2VLJD                 45    0.024   4.339   1.191   0.011   0.918   0.071
B V  2VLJD                 46    0.026   4.058   0.476   0.001   0.959   0.040
B T  2VLJD                 47    0.081  11.235   0.308   0.001   0.959   0.040
B V  2VLJD                 48    0.093  14.325  -0.009   0.001   0.959   0.040
B V  2VLJD                 49    0.170  26.190  -0.465   0.003   0.718   0.279
B T  2VLJD                 50    0.255  35.396  -1.033   0.004   0.420   0.576
E G  2VLJD                 51    0.646  50.832  -1.426   0.018   0.088   0.893
E G  2VLJD                 52    0.486  38.225  -0.886   0.018   0.047   0.935
E E  2VLJD                 53    0.651 113.765  -0.843   0.018   0.088   0.893
E V  2VLJD                 54    0.662 101.765  -0.556   0.019   0.141   0.840
E K  2VLJD                 55    0.645 132.615  -0.240   0.019   0.141   0.840
E K  2VLJD                 56    0.392  80.676   0.238   0.018   0.088   0.893
E L  2VLJD                 57    0.664 121.505  -0.796   0.004   0.085   0.910
E K  2VLJD                 58    0.476  97.934  -1.535   0.004   0.085   0.910
B R  2VLJD                 59    0.267  61.097  -0.314   0.004   0.420   0.576
B L  2VLJD                 60    0.054   9.887   0.054   0.003   0.718   0.279
E T  2VLJD                 61    0.278  38.545   0.163   0.001   0.900   0.099
B F  2VLJD                 62    0.178  35.785  -2.378   0.001   0.900   0.099
E Q  2VLJD                 63    0.413  73.690  -0.253   0.001   0.900   0.099
B F  2VLJD                 64    0.259  52.062  -0.861   0.002   0.816   0.182
E G  2VLJD                 65    0.426  33.558  -0.190   0.004   0.616   0.381
E D  2VLJD                 66    0.536  77.266  -0.813   0.004   0.197   0.799
E A  2VLJD                 67    0.665  73.305  -0.882   0.004   0.085   0.910
E R  2VLJD                 68    0.598 136.942  -0.547   0.004   0.085   0.910
E K  2VLJD                 69    0.393  80.861  -0.196   0.005   0.336   0.660
E D  2VLJD                 70    0.339  48.792   0.525   0.002   0.816   0.182
B S  2VLJD                 71    0.046   5.426  -0.304   0.001   0.900   0.099
B S  2VLJD                 72    0.212  24.823   0.769   0.001   0.959   0.040
B L  2VLJD                 73    0.027   4.870   0.595   0.001   0.959   0.040
B H  2VLJD                 74    0.253  46.021   0.569   0.001   0.959   0.040
B I  2VLJD                 75    0.053   9.879   0.384   0.001   0.900   0.099
E T  2VLJD                 76    0.467  64.745   0.689   0.002   0.816   0.182
E A  2VLJD                 77    0.380  41.865  -0.582   0.004   0.514   0.481
B A  2VLJD                 78    0.152  16.783  -0.300   0.005   0.262   0.733
E Q  2VLJD                 79    0.453  80.959   0.526   0.004   0.197   0.799
E P  2VLJD                 80    0.435  61.755  -0.393   0.020   0.205   0.775
E G  2VLJD                 81    0.530  41.719  -0.929   0.018   0.088   0.893
B D  2VLJD                 82    0.224  32.250  -0.803   0.018   0.088   0.893
B T  2VLJD                 83    0.170  23.593  -0.273   0.022   0.359   0.619
B G  2VLJD                 84    0.049   3.848  -0.866   0.003   0.718   0.279
B L  2VLJD                 85    0.130  23.803   0.604   0.001   0.900   0.099
B Y  2VLJD                 86    0.032   6.838   0.738   0.001   0.900   0.099
B L  2VLJD                 87    0.045   8.221   0.848   0.001   0.900   0.099
B C  2VLJD                 88    0.015   2.162   1.313   0.001   0.900   0.099
B A  2VLJD                 89    0.031   3.405  -0.175   0.002   0.816   0.182
B G  2VLJD                 90    0.080   6.272  -0.521   0.003   0.718   0.279
B A  2VLJD                 91    0.155  17.114  -0.123   0.004   0.514   0.481
E G  2VLJD                 92    0.408  32.110  -1.220   0.004   0.197   0.799
E S  2VLJD                 93    0.536  62.784  -2.142   0.004   0.085   0.910
E Q  2VLJD                 94    0.436  77.905  -1.798   0.005   0.045   0.951
E G  2VLJD                 95    0.363  28.568  -1.130   0.018   0.047   0.935
E N  2VLJD                 96    0.351  51.357  -0.065   0.019   0.141   0.840
B L  2VLJD                 97    0.144  26.275   0.468   0.004   0.420   0.576
B I  2VLJD                 98    0.048   8.824  -0.538   0.023   0.655   0.322
B F  2VLJD                 99    0.065  13.045  -1.147   0.021   0.756   0.223
B G  2VLJD                100    0.052   4.053  -3.689   0.004   0.514   0.481
E K  2VLJD                101    0.398  81.827  -0.320   0.004   0.138   0.858
E G  2VLJD                102    0.270  21.233  -1.298   0.005   0.045   0.951
E T  2VLJD                103    0.416  57.658  -0.220   0.004   0.138   0.858
E K  2VLJD                104    0.556 114.307  -0.013   0.004   0.514   0.481
B L  2VLJD                105    0.135  24.700   0.040   0.022   0.552   0.426
E S  2VLJD                106    0.382  44.794  -0.220   0.023   0.655   0.322
B V  2VLJD                107    0.275  42.329  -1.561   0.023   0.655   0.322
E K  2VLJD                108    0.432  88.904  -0.611   0.022   0.552   0.426
E P  2VLJD                109    0.463  65.671  -2.340   0.022   0.359   0.619
E N  2VLJD                110    0.525  76.831  -1.215   0.020   0.205   0.775
E I  2VLJD                111    0.434  80.235  -1.253   0.018   0.088   0.893
E Q  2VLJD                112    0.566 101.177  -1.218   0.018   0.088   0.893
E N  2VLJD                113    0.551  80.593  -1.207   0.018   0.047   0.935
B P  2VLJD                114    0.270  38.384  -0.704   0.018   0.019   0.964
E D  2VLJD                115    0.564  81.287  -0.510   0.018   0.019   0.964
B P  2VLJD                116    0.190  26.918  -2.156   0.018   0.088   0.893
E A  2VLJD                117    0.286  31.473  -1.540   0.022   0.359   0.619
B V  2VLJD                118    0.255  39.193  -1.721   0.022   0.552   0.426
B Y  2VLJD                119    0.268  57.293  -1.202   0.021   0.451   0.528
E Q  2VLJD                120    0.354  63.207  -0.828   0.021   0.279   0.699
B L  2VLJD                121    0.137  25.066  -2.495   0.004   0.197   0.799
E R  2VLJD                122    0.418  95.653  -0.543   0.004   0.085   0.910
E D  2VLJD                123    0.517  74.442  -0.387   0.005   0.045   0.951
E S  2VLJD                124    0.561  65.808  -1.736   0.005   0.015   0.979
E K  2VLJD                125    0.762 156.641  -1.187   0.016   0.005   0.979
E S  2VLJD                126    0.425  49.775  -1.448   0.018   0.019   0.964
E S  2VLJD                127    0.506  59.327  -0.036   0.004   0.138   0.858
B D  2VLJD                128    0.188  27.163  -0.102   0.004   0.616   0.381
B K  2VLJD                129    0.111  22.750   0.158   0.002   0.816   0.182
B S  2VLJD                130    0.042   4.922  -0.216   0.001   0.900   0.099
B V  2VLJD                131    0.032   4.903   0.380   0.011   0.918   0.071
B C  2VLJD                132    0.017   2.373   1.361   0.011   0.918   0.071
B L  2VLJD                133    0.041   7.470   0.217   0.018   0.846   0.136
B F  2VLJD                134    0.045   9.011  -0.055   0.003   0.718   0.279
B T  2VLJD                135    0.203  28.212   0.565   0.004   0.420   0.576
E D  2VLJD                136    0.484  69.744  -1.275   0.004   0.138   0.858
B F  2VLJD                137    0.161  32.252  -0.542   0.004   0.085   0.910
E D  2VLJD                138    0.285  40.996   0.134   0.004   0.085   0.910
B S  2VLJD                139    0.213  24.940  -1.145   0.004   0.085   0.910
E Q  2VLJD                140    0.619 110.482  -0.520   0.004   0.085   0.910
E T  2VLJD                141    0.463  64.246  -1.313   0.005   0.262   0.733
B N  2VLJD                142    0.109  15.987   0.053   0.003   0.718   0.279
E V  2VLJD                143    0.372  57.238   0.143   0.001   0.900   0.099
B S  2VLJD                144    0.065   7.653  -0.567   0.001   0.900   0.099
E Q  2VLJD                145    0.336  59.992   0.750   0.001   0.959   0.040
B S  2VLJD                146    0.153  17.943   0.097   0.001   0.900   0.099
E K  2VLJD                147    0.393  80.737   0.703   0.002   0.816   0.182
E D  2VLJD                148    0.475  68.419  -0.672   0.004   0.514   0.481
E S  2VLJD                149    0.717  83.997  -1.439   0.005   0.045   0.951
E D  2VLJD                150    0.662  95.322  -1.767   0.004   0.085   0.910
E V  2VLJD                151    0.511  78.587  -0.480   0.004   0.616   0.381
E Y  2VLJD                152    0.511 109.222  -0.190   0.003   0.718   0.279
E I  2VLJD                153    0.321  59.385  -0.145   0.004   0.616   0.381
E T  2VLJD                154    0.503  69.794  -1.231   0.004   0.514   0.481
E D  2VLJD                155    0.539  77.728  -1.388   0.005   0.262   0.733
E K  2VLJD                156    0.532 109.391  -1.105   0.005   0.262   0.733
E T  2VLJD                157    0.309  42.928  -1.227   0.003   0.718   0.279
E V  2VLJD                158    0.401  61.618  -0.416   0.001   0.900   0.099
E L  2VLJD                159    0.346  63.334  -0.238   0.001   0.900   0.099
E D  2VLJD                160    0.353  50.882   0.075   0.021   0.756   0.223
E M  2VLJD                161    0.281  56.228  -0.153   0.004   0.616   0.381
E R  2VLJD                162    0.547 125.194  -1.070   0.005   0.336   0.660
E S  2VLJD                163    0.636  74.492  -1.762   0.004   0.197   0.799
E M  2VLJD                164    0.530 106.053  -1.840   0.004   0.138   0.858
E D  2VLJD                165    0.566  81.633  -1.881   0.004   0.197   0.799
E F  2VLJD                166    0.315  63.160  -2.274   0.004   0.420   0.576
B K  2VLJD                167    0.268  55.045  -0.563   0.003   0.718   0.279
B S  2VLJD                168    0.175  20.451  -1.381   0.002   0.816   0.182
B N  2VLJD                169    0.156  22.780   0.054   0.001   0.900   0.099
B S  2VLJD                170    0.159  18.658  -0.810   0.002   0.816   0.182
B A  2VLJD                171    0.122  13.433  -1.707   0.018   0.846   0.136
B V  2VLJD                172    0.137  21.072  -2.157   0.011   0.918   0.071
E A  2VLJD                173    0.292  32.211  -0.058   0.001   0.900   0.099
B W  2VLJD                174    0.172  41.318  -0.578   0.004   0.616   0.381
E S  2VLJD                175    0.454  53.185   0.433   0.004   0.085   0.910
E N  2VLJD                176    0.748 109.434   0.019   0.005   0.015   0.979
E K  2VLJD                177    0.743 152.815   0.214   0.005   0.015   0.979
E S  2VLJD                178    0.621  72.781   0.517   0.004   0.138   0.858
E D  2VLJD                179    0.533  76.748   1.358   0.004   0.616   0.381
B F  2VLJD                180    0.104  20.833   1.039   0.001   0.900   0.099
E A  2VLJD                181    0.445  49.006   1.873   0.001   0.959   0.040
B C  2VLJD                182    0.032   4.479  -0.110   0.001   0.959   0.040
E A  2VLJD                183    0.422  46.460   1.778   0.011   0.918   0.071
B N  2VLJD                184    0.111  16.177  -0.032   0.011   0.918   0.071
E A  2VLJD                185    0.529  58.307   1.089   0.001   0.900   0.099
B F  2VLJD                186    0.211  42.247   0.603   0.003   0.718   0.279
E N  2VLJD                187    0.715 104.735   0.547   0.005   0.045   0.951
E N  2VLJD                188    0.778 113.885  -0.315   0.003   0.003   0.994
E S  2VLJD                189    0.720  84.384  -0.334   0.004   0.085   0.910
E I  2VLJD                190    0.490  90.687  -0.329   0.004   0.420   0.576
E I  2VLJD                191    0.410  75.813   0.532   0.004   0.514   0.481
E P  2VLJD                192    0.396  56.178   0.092   0.022   0.552   0.426
E E  2VLJD                193    0.486  84.957  -1.296   0.021   0.451   0.528
E D  2VLJD                194    0.599  86.345  -0.424   0.005   0.336   0.660
E T  2VLJD                195    0.548  75.952  -0.002   0.004   0.420   0.576
B F  2VLJD                196    0.205  41.063  -1.550   0.004   0.420   0.576
E F  2VLJD                197    0.549 110.164  -0.429   0.005   0.262   0.733
E P  2VLJD                198    0.392  55.667  -1.519   0.004   0.085   0.910
E S  2VLJD                199    0.871 102.093  -2.292   0.003   0.003   0.994

Explain the output.  Go back.
```

---
